# Factors Associated With Home Births in Benin and Mali: Evidence From the Recent Demographic and Health Surveys

**DOI:** 10.3389/frph.2022.808070

**Published:** 2022-02-17

**Authors:** Aristide Romaric Bado, Hermann Badolo, Ermel Johnson, Evelyne Bewendin Komboigo, Sètondji Géraud R. Padonou, Fatou Diawara

**Affiliations:** ^1^Département Biomedical et Santé Publique, Institut de Recherche en Sciences de la Santé (IRSS), Ouagadougou, Burkina Faso; ^2^Department of Public Health and Research, West African Health Organization, Bobo Dioulasso, Burkina Faso; ^3^Observatoire de la Population, Institut National de Santé Publique (INSP), Ouagadougou, Burkina Faso; ^4^Department of Statistics and Population Stadies, University of the Western Cape, Cape Town, South Africa; ^5^Sourou Sanou University Hospital (CHUSS), Bobo-Dioulasso, Burkina Faso; ^6^Département de Santé Publique, Faculté des Sciences de la Santé (FSS), Université d'Abomey-Calavi (UAC), Cotonou, Benin; ^7^Département Études et Recherches Médicale et Communautaire, Institut National de Santé Publique, Bamako, Mali

**Keywords:** determinants of homebirth, Mali, Benin, DHS data, sub-Saharan Africa

## Abstract

**Introduction:**

Identifying and understanding the factors associated with homebirths can contribute to improving maternal and child health and achieving the Sustainable Development Goals (SDGs). This study aimed to perform a comparative analysis of the factors associated with homebirths in Benin and Mali.

**Method:**

This study is based on the most recent data from the Demographic Health Surveys conducted in Mali and Benin in 2018. The dependent variable was homebirth, and the explanatory variables were the individual characteristics of the woman, the distance to the health center, the place of residence, the number of prenatal consultations had, the frequency of media exposure, and the use of the Internet. The primary survey unit (PSU) was considered in the analysis to measure the effect of context on the choice of the place of delivery. Further, descriptive statistics and multilevel logistic regression analysis were used in the study.

**Results:**

Educational level was associated with homebirth in Benin and Mali; Women with either no education or primary education are more likely to give birth at home. Women who didn't live close to a health facility were more likely to give birth at home than those who didn't face this problem in both countries. Not making visits for antenatal care (ANC) increases the odds of having a homebirth by 31.3 times (CI = 24.10–40.70) in Benin and 12.91 times (CI = 10.21–16.33) in Mali. Similarly, women who went on 1–2 ANC visits were more likely to give birth at home compared with women who made five or more ANC visits in both countries. The number of children per woman was also a significant factor in both countries. Women who often or regularly paid attention to the media messages were less likely to give birth at home compared with those who did not follow relevant media inputs (aOR = 0.42 [CI = 0.26–0.67] in Benin and aOR = 0.65 [CI = 0.50–0.85] in Mali).

**Conclusion:**

Increasing the demand and uptake of women's health services by improving the availability and quality of services and establishing community health centers could help reduce the incidence of homebirths that can be risky and, thus, combat maternal and infant mortality.

## Introduction

The reduction of maternal mortality and morbidity is an important goal on the health agenda of international, regional, and national organizations/programmes. Since the Nairobi International Conference on Safe Motherhood in 1987, the international community has become aware of the seriousness of maternal mortality in the world, especially in developing countries (1). The earnest pursuit of lowering the incidence of maternal mortality has been further affirmed at international summits. Indeed, at the Millennium Summit in September 2000, the world's heads of state set themselves the goal of reducing maternal mortality worldwide by 75% by 2015 (2). Similarly, the Sustainable Development Goals (SDGs) have placed a strong emphasis on decreasing maternal mortality by aiming to reduce the global maternal mortality ratio (MMR) to below 70 per 100,000 live births by 2030 and ensuring universal access to sexual and reproductive healthcare services, including family planning, information and education, and the integration of reproductive health into national strategies and programmes (3). Maternal health issues are one of the world's major health challenges; however, over the past three decades, significant progress has been made resulting in a 40% decline in the global MMR between 2010 and 2014 (4). Despite this progress, worldwide, 295,000 women died because of pregnancy and childbirth in 2017 (2). Notably, sub-Saharan Africa has the highest maternal mortality rate among the seven regions of the world, with 534 deaths per 100,000 live births (2).

Homebirth is one of the main reasons for the high maternal mortality rate in sub-Saharan Africa (5). Sub-Saharan Africa and South Asia together contribute to more than 85% of maternal deaths. Of these, only half of the deliveries are institutional (6). There is growing evidence that high MMR in sub-Saharan Africa is strongly linked to homebirth as most of the births in this region occurs at home (7). Homebirths, more so in the absence of trained professional attendants, have been associated with adverse infant and maternal outcomes (8, 9). Institutional delivery is considered the most critical intervention to reduce maternal mortality and ensure safe motherhood (10). The World Health Organization (WHO) recommends that every delivery be supervised by a skilled attendant, a health professional who can identify and manage normal labor and delivery, identify and treat complications, and/or provide basic care and referral (11). However, the proportion of births attended by skilled health personnel is still below the recommended levels (12) despite national efforts to increase the availability of maternal health services (13) and implement programmes to subsidize normal deliveries and/or cesarean sections in some countries (14, 15).

In West Africa, many women give birth without the assistance of a skilled health professional; according to the results of the Demographic and Health Surveys (DHSs), homebirth rates were 59 in Nigeria (2018) (16), 47.3 in Guinea (2018) (17), 33% in Mali (2018) (18), 16 in Sierra Leone (2019) (19), and 14.5% in Benin (2018) (20). Such high proportions of homebirth are associated with high maternal mortality and morbidity and under-five mortality in these countries (21). Identifying and understanding the factors related to home births can, therefore, contribute to improving maternal and child health and achieving the related SDG. Benin and Mali are two French-speaking countries in the Economic Community of West African States (ECOWAS) that have implemented Emergency Obstetric and Neonatal Care (EmONC) policies to alleviate the economic burden on women during childbirth. These two countries also conducted a DHS in 2018 that collected recent data to study the determinants associated with homebirth. This study performed a comparative analysis of the factors associated with homebirths in Benin and Mali.

## Methods

### Study Area

The two countries examined in this study (Republic of Benin and Republic of Mali) are in West Africa. The population of Benin is estimated by the United Nations to be 12.4 million in 2021^1^. The average number of children born per woman of childbearing age was 5.7 in 2017–2018 (20). Benin's Human Development Index (HDI) for 2019 was 0.545, which places the country in the 158th position out of 189 countries and territories. According to the results of the 2018 Integrated Regional Survey on Employment and the Informal Sector, nearly seven out of ten school-age children were enrolled in school, and the literacy rate among those aged 15 years or olderwas 41.7% (22). Mali's population is estimated by the United Nations to be 20.9 million in 2021 (1), 51% of which are women. The total fertility rate remains high in Mali, with an average of 6.3 children per woman (18). Three-quarters of the population lives in rural areas (74.5%)^2^, and the population is unevenly dispersed over a vast territory of 1,240,192 km^2^. Mali's HDI for 2019 was 0.434, placing it in the ‘low human development' category. In Mali, in 2016, nearly six out of ten children (60.2%) aged 7–12 did not attend the first cycle of basic education, and only 31.0% of adults were literate (23).

At the health level, Benin's health system is pyramidal with three levels: the central or national level constitutes the Ministry of Health, its programmes, and the national hospitals; the intermediate or departmental level includes the departmental health directorates, their services, and the departmental hospitals; and the operational or peripheral level is represented by the health zones (24). Each health zone comprises one to four communes. The health zone is further divided into health areas that group together villages or neighborhoods (25).

The Malian health system is structured on the following three levels: (i) the operational level of technical support to the community health centers, consisting of the district management team; (ii) the intermediate regional level of technical support, made up of the Regional Health Directorates; and (iii) the strategic national level, comprising the Minister's office, the General Secretariat, and the central services of the Ministry of Health and Public Hygiene of Mali (26). The health services include all the public (state and local authorities), private, community (associations, mutual societies, foundations, etc.), and denominational structures and bodies, as well as the professional health orders, whose action contributes to the implementation of the national health policy.

### Data Sources

The study employed the most recent data from the DHSs conducted in Mali and Benin in 2018. The sample for this study included women aged 15–49 years who gave birth in the five years preceding a survey in each of the two countries conducted for this study. A total of 15,362 and 10,519 women were interviewed in the survey in Benin and Mali, respectively, while only 8,994 in Benin and 6,368 in Mali were eligible for the current study. These women were those aged 15–49 years who had a live birth in the 5 years preceding the survey in Benin and Mali.

The DHS was conducted using a two-stage cluster sampling design, based on enumeration areas and household samples. The detailed methodology of the surveys was described in the final reports (18, 20). The DHSes are nationally representative, cross-sectional surveys that collect information on a wide range of public health topics, such as anthropometric, demographic, socio-economic, family planning, and domestic violence data, to name a few. The survey covered men and women aged 15–49 years and children less than 5 years of age residing in non-institutional settings. The DHS datasets are available to researchers on the DHS website at: http://dhsprogram.com/data/available-datasets.cfm.

## Variables

### Variable Outcome

The outcome variable was homebirth, which was generated from the variable ‘place of birth' assigned as 1 if the birth occurred at home and 0 if birth occurred in another place (hospital/clinic/health facility/other). This variable was collected from women based on self-reports.

### Independent Variables

The explanatory variables were age, level of education, distance to a health center, place of residence, the household's standard of living, occupation of the woman, number of antenatal visits made, number of children of the woman, experience of an interrupted pregnancy, sex of the head of the household, the spouse's level of education and occupation, the frequency of media exposure and internet use. These variables were considered because of their statistically significant relationships with the place of delivery observed in previous studies and their availability in the dataset. The primary survey unit (PSU) was considered in the analyses to measure the effect of context on the choice of delivery location.

### Statistical Analyses

We analyzed the collected data using Stata version 16.0. The analyses included both descriptive statistics (frequencies and percentages) to describe the respondents' characteristics and multilevel logistic regression analyses to identify the determinants. We used multivariate multilevel logistic regression to determine the individual and community-level factors that could affect homebirth. As the DHS data are hierarchical, i.e., mothers are nested within households and households are nested within clusters (PSUs), the use of the multilevel model is therefore appropriate to more accurately identify the factors associated. Two-level multilevel logistic regression model (individual and community factors). The household level was not retained because the probability of having more than one woman giving birth is low. We ran two models: the empty model and the model combining individual and community factors. The results were presented as adjusted odds ratios (aOR), with the corresponding 95% confidence intervals (CI) indicating their level of precision. Statistical significance was reported at *p* < 0.05. Intraclass correlations (ICCs) were used to measure intraclass variables (27).

## Ethical Considerations

This study is based on the analysis of secondary data without using any identified information about the participant. All DHSs were approved by ICF International and a national ethics committee in each host country. All participants gave informed written consent before taking part in the survey. In this study, additional ethics approval was not required, but we did obtain written permission from the DHS Program to use the data.

## Results

### Descriptive Analyses and Chi-Square Test Results

Table 1 presents the results on homebirths according to the explanatory variables considered in this study for Benin and Mali. The proportion of homebirths is 13.7 in Benin and 32.9% in Mali. The results of the chi-square test show that the variables with a statistically significant association with homebirth are almost the same in both countries. In Benin, the women's level of education, marital status, problem of distance to a health center, place of residence, household' standard of living, the woman's occupation, number of prenatal visits made, number of children the woman has, the experience of an interrupted pregnancy, gender of the head of household, relationship with the head of household, education and occupation of the spouse, frequency of media exposure and internet use are the variables that were found to be linked to the place of delivery. The same variables (except the experience of an aborted pregnancy) are associated with the dependent variable in Mali. In both countries, no education (19 in Benin and 40% in Mali), having a problem of distance to a health center (24 in Benin and 52.3% in Mali), not having a prenatal consultation (68.2 in Benin and 74.1% in Mali), women having seven or more children (22.5 in Benin and 37.6% in Mali), having an uneducated spouse (22.5 in Benin and 40.4% in Mali), the frequency of paying attention to the media (21.1 in Benin and 50.8% in Mali), and not using the Internet (14.4 in Benin and 36.1% in Mali) are the factors that predispose a woman to homebirth.

**Table 1:** Descriptive and chi-square test results.

**Variables**	**Benin**				**Mali**			
	***n* (%)**	**Home delivery**		***P* value**	***n* (%)**	**Home delivery**		***P* value**
		**No (%)**	**Yes (%)**			**No (%)**	**Yes (%)**	
**Age**								
15–24	2,407 (26.8)	87.2	12.8	0.101	2,021 (31.7)	68.6	31.4	0.146
25–34	4,372 (48.6)	86.4	13.6		2,854 (44.8)	66.8	33.2	
35–49	2,215 (24.6)	85.1	14.9		1,493 (23.4)	65.6	34.4	
**Mother's education**								
No education	5,786 (64.3)	81	19		4,547 (71.4)	59.4	40.6	0.001
Primary	1,622 (18.0)	93.3	6.7		797 (12.5)	78	22	
Secondary +	1,586 (17.6)	98.2	1.8		1,024 (16.1)	92.5	7.5	
**Current marital status**								
Never in union	302 (3.4)	92.4	7.6	0.000	169 (2.7)	79.9	20.1	0.001
Married	6,607 (73.5)	85.5	14.5		6,034 (94.8)	66.9	33.1	
Living with partner	1,724 (19.2)	87.8	12.2		32 (0.5)	68.8	31.2	
Widowed/divorced/separated	361 (4.0)	88.4	11.6		133 (2.1)	60.2	39.8	
**Problem of distance to a health center**								
Big problem	3,024 (33.6)	76	24	0.001	2,048 (32.2)	47.7	52.3	0.001
Not a big problem	5,970 (66.4)	91.5	8.5		4,320 (67.8)	76.3	23.7	
**Residency**								
Urban	3,636 (40.4)	91.8	8.2	0.001	1,726 (27.1)	83.1	16.9	0.001
Rural	5,358 (59.6)	82.5	17.5		4,642 (72.9)	61.1	38.9	
**Household wealth index**								
Poorest	1,889 (21.0)	65.5	34.5	0.001	1,190 (18.7)	43.0	57.0	0.001
Poorer	1,810 (20.1)	82.8	17.2		1,260 (19.8)	52.4	47.6	
Middle	1,773 (19.7)	88.8	11.2		1,334 (20.9)	62.8	37.2	
Richer	1,785 (19.9)	96.3	3.7		1,361 (21.4)	81.6	18.4	
Richest	1,737 (19.3)	99.6	0.4		1,223 (19.2)	94.2	5.8	
**Women's occupation**								
Not Working	1,538 (17.1)	83	17	0.001	2,901 (45.6)	62.8	37.2	0.001
Professional/technical/managerial/clerical	440 (4.9)	94.1	5.9		166 (2.6)	94	6	
Agricultural & Self Employed	3,144 (35.0)	79.2	20.8		1,442 (22.6)	63	37	
Sales	3,872 (43.1)	92.5	7.5		1,859 (29.2)	74.6	25.4	
**Number of antenatal care (ANC) visits during pregnancy**								
0 visits	1,030 (11.5)	31.8	68.2	0.001	1,432 (22.5)	25.9	74.1	0.001
1–2 visits	1,646 (18.3)	84.2	15.8		927 (14.6)	62	38	
3–4 visits	1,476 (16.4)	93.2	6.8		1,195 (18.8)	74.6	25.4	
5 and more	4,842 (53.8)	96.5	3.5		2,814 (44.2)	86.5	13.5	
**Number of children**								
01–02	3,366 (37.4)	89.8	10.2	0.001	2,210 (34.7)	72.4	27.6	0.001
03–04	2,793 (31.1)	87.1	12.9		1,819 (28.6)	65.5	34.5	
05–06	1,679 (18.7)	83.9	16.1		1,268 (19.9)	64.2	35.8	
7&+	1,156 (12.9)	77.5	22.5		1,071 (16.8)	62.4	37.6	
**Ever had a terminated pregnancy**								
Yes	1,097 (12.2)	90.7	9.3	0.001	747 (11.7)	68	32	0.569
No	7,897 (87.8)	85.7	14.3		5,621 (88.3)	67	33	
**Sex of the household head**								
Male	7,471 (83.1)	85	15	0.001	5,512 (86.6)	67.8	32.2	0.002
Female	1,523 (16.9)	92.5	7.5		856 (13.4)	62.5	37.5	
**Relationship to household head**								
Wife	5,325 (59.2)	87.5	12.5	0.001	4,746 (74.5)	66.8	33.2	
Head	893 (9.9)	91	9		592 (9.3)	61.1	38.9	
Daughter	630 (7.0)	91.4	8.6		299 (4.7)	69.9	30.1	
Daughter-in-Law	780 (8.7)	81.2	18.8		383 (6.0)	70.2	29.8	
Other relative	1,366 (15.2)	79	21		348 (5.5)	75	25	
**Husband/partner's educational attainment**								
No education	4,373 (48.6)	77.5	22.5	0.001	4,332 (68.0)	59.6	40.4	0.001
Primary	1,585 (17.6)	93.3	6.7		552 (8.7)	76.1	23.9	
Secondary & +	1,977 (22.0)	97.1	2.9		967 (15.2)	90.6	9.4	
Not precised	396 (4.4)	94.4	5.6		215 (3.4)	83.7	16.3	
Missing information	663 (7.4)	90.2	9.8		302 (4.7)	71.2	28.8	
**Husband occupation**								
Not Working	1,666 (18.5)	83.1	16.9	0.001	2,946 (46.3)	63.2	36.8	0.001
Professional/technical/managerial	295 (3.3)	98.3	1.7		121 (1.9)	94.2	5.8	
Sales	2,806 (31.2)	91.6	8.4		1,489 (23.4)	78.4	21.6	
Agricultural - self employed	2,168 (24.1)	72.8	27.2		1,441 (22.6)	63	37	
Services/Skilled manual	2,059 (22.9)	94	6		371 (5.8)	59.3	40.7	
**Sex of household head**								
Male	7,471 (83.1)	85	15	0.001	5,512 (86.6)	67.8	32.2	0.002
Female	1,523 (16.9)	92.5	7.5		856 (13.4)	62.5	37.5	
**Frequency of media exposure (radio, tv, magazines)**								
Not at all	3,518 (39.1)	78.9	21.1	0.001	1,370 (21.5)	49.2	50.8	0.001
Often	4,211 (46.8)	89.1	10.9		3,230 (50.7)	66.1	33.9	
Regularly	1,265 (14.1)	97.6	2.4		1,768 (27.8)	82.8	17.2	
**Use of internet**								
Never	8,528 (94.8)	85.6	14.4	0.001	5,603 (88.0)	63.9	36.1	0.001
Yes	466 (5.2)	99.5	0.5		765 (12.0)	92.1	7.9	
***N***	**8,994 (100)**	**86.3**	**13.7**		**6,368 (100)**	**67.1**	**32.9**	

## Results of the Multivariate Multilevel Logistic Regression Analysis

### Measures of Variation (Random Effects)

As shown in Table 2, Model 0 (empty model), there is significant variation in the probability of giving birth at home across PSUs in both Benin and Mali (*p* < 0.001). According to the ICC, the act of giving birth at home could be attributed, respectively to factors linked to the PSUs, which in several cases correspond to villages in the rural environment and individual factors. The variations between the PSUs remained statistically significant, even after controlling for all the factors in Model 1 (with all the independent variables) in Benin and Mali.

**Table 2:** Multilevel logistic regression analysis on the predictors of home deliveries in Benin and Mali.

**Variables**	**Benin**		**Mali**	
	**Model 0**	**Model 1**	**Model 0**	**Model 1**
		**Odds ratio (95% CI)**		**Odds ratio (95% CI)**
**Fixed effects**				
**Age**				
15–24		1		1
25–34		1.05 (0.80; 1.36)		0.74 (0.58; 0.94)**
35–49		0.97 (0.68; 1.39)		0.66 (0.48; 0.91)**
**Mother's education**				
No education		2.52 (1.59; 4.00)***		2.21 (1.58; 3.10)***
Primary		2.08 (1.27; 3.43)**		1.42 (0.96; 2.10)
Secondary +		1		1
**Current marital status**				
Never in union		1		1
Married		0.54 (0.27; 1.04)		0.93 (0.48; 1.81)
Living with partner		0.98 (0.49; 1.94)		3.85 (1.09; 13.51)**
Widowed/divorced/separated		0.54 (0.25; 1.17)		1.25 (0.56; 2.80)
**Problem of distance to a health center**				
Big problem		1.27 (1.05; 1.54)**		1.32 (1.10; 1.59)**
Not a big problem		1		1
**Residency**				
Urban		1		1
Rural		1.62 (1.14; 2.30)**		1.76 (1.05; 2.98)***
**Household wealth index**				
Poorest		14.5 (6.1; 34.40)***		3.63 (2.16;6.12)***
Poorer		11.8 (5.0; 27.9)***		3.1 (1.85; 5.14)***
Middle		9.6 (4.1; 22.6)***		2.74 (1.67; 4.51)***
Richer		5.2 (2.2; 1.5)***		1.78 (1.17; 2.72)**
Richest		1		1
**Women's occupation**				
Not Working		1		1
Professional/technical/managerial/clerical		1.13 (0.54; 2.33)		0.34 (0.07; 1.48)
Agricultural & Self Employed		0.59 (0.08; 3.93)		0.006(0.001; 0.009)
Sales		0.45 (0.06; 3.16)		1.10 (0.74; 1.64)
**Number of antenatal care (ANC) visits during pregnancy**				
0 visits		31.32 (24.10; 40.70)***		12.91 (10.21; 16.33)***
1–2 visits		3.07 (2.41; 3.91)***		2.37 (1.87; 3.00)***
3–4 visits		1.40 (1.05; 1.86)**		1.42 (1.14; 1.78)**
5 and more		1		1
**Number of children**				
01–02		0.59 (0.41; 0.84)**		0.58 (0.42; 0.82)**
03–04		0.71 (0.52; 0.97)**		0.94 (0.71; 1.26)
05–06		0.84 (0.62; 1.12)		0.96 (0.73; 1.26)
7&+		1		1
**Ever had a terminated pregnancy**				
Yes		1		1
No		1.00 (0.74; 1.35)		1.13 (0.88; 1.45)
**Sex of the household head**				
Male		1		1
Female		0.67 (0.41; 1.09)		1.47 (0.92; 2.37)
**Relationship to household head**				
Wife		1		1
Head		1.62 (0.91; 2.88)		0.76 (0.44; 1.30)
Daughter		0.84 (0.52; 1.34)		0.60 (0.36; 1.00)
Daughter-in-Law		1.30 (0.95; 1.78)		1.14 (0.77; 1.66)
Other relative		1.31 (1.04;1.66)**		0.68 (0.43; 1.07)
**Husband occupation**				
No education		1		1
Professional/technical/managerial		0.94 (0.26; 3.36)		1.46 (0.24; 8.89)
Sales		2.12 (029; 15.22)		0.72 (0.47; 1.11)
Agricultural - self employed		2.48 (0.37; 16.57)		71629.58
Services/Skilled manual		1.70 (0.24; 11.79)		1
**Frequency of media exposure (radio, tv, magazines)**				
Not at all		1		1
Often		0.77 (0.64; 0.93)**		0.91 (0.74; 1.11)
Regularly		0.42 (0.26; 0.67)***		0.65 (0.50; 0.85)**
**Use of Internet**				
Never		1		1
Yes		0.38 (0.11; 1.34)		0.87 (0.60; 1.26)
**Constant**	−3.60 (−3,89; −3.31)	0.017 (0.006; 0.044)***	−1.35 (−1.63; −1.08)***	0.045 (0.017; 0.117)***
**Random effects**				
Primary sampling unit (PSU)	5.00 (4.01; 6.23)	1.65 (1.23; 2.20)	5.52 (4.47; 6.80)	2.93 (2.33; 3.69)
Intra-cluster Correlation Coefficient (ICC (95% CI))	0.603 (0.549; 0.654)	0.333 (0.272; 0.401)	0.626 (0.576; 0.674)	0.472 (0.415; 0.529)
**Log likelihood**	–2677.16***	–2114.39***	–2798.55***	–2408.92***

**p-value < 0.05, ***p-value < 0.01.

### Measures of Association (Fixed Effects)

Table 2 presents the results regarding the factors associated with homebirth among women in Benin and Mali. The probability of giving birth at home is higher among women with no education (aOR = 2.52, 95%CI = 1.59–4.00) and among women with primary education (aOR = 2.08, 95%CI = 1.27–3.43) compared with women having secondary education or above in Benin. The same trend is observed in Mali, and the aORs are 2.21 (95%CI = 1.58–3.10) and 1.42 (95%CI = 0.96–2.10), respectively for women with no education and those with primary education.

In both countries, women experiencing distance problems to a health facility are more likely to give birth at home than those who do not face this problem. In Benin, women experiencing the problem of distance to a health center are 1.27 times (95%CI = 1.05–1.54) more likely to give birth at home. This risk is 1.32 times (95%CI = 1.10–1.59) for women facing the same problem in Mali.

Women residing in rural areas in Benin [aOR = 1.62 (95%CI = 1.14–2.30)] and Mali [aOR = 3.20 (95%CI = 1.97–5.22)] were more likely to give birth at home compared with those residing in urban areas. In both countries, the standard of living of the household is significant – women from poor households are more likely to give birth at home than women from better-off households.

In Benin and Mali, the odds of giving birth at home is strongly related to the number of antenatal visits a woman makes during her pregnancy. Not having made any ANC visits increases the odds of having a homebirth by 31.3 (aOR=31.3; 95%CI = 24.10–40.70) in Benin and 12.91 (95%CI = 10.21–16.33) in Mali. Similarly, women who went on 1–2 antenatal visits were more likely (aOR = 3.07 (95%CI = 2.41–3.91) in Benin and aOR = 2.37 (95%CI = 1.87–3.00) in Mali) to give birth at home than women who made five or more antenatal visits. Furthermore, the risk of giving birth at home was 1.4 times (95%CI = 1.05–1.86) higher in Benin and 1.42 times (95%CI = 1.14–1.78) higher in Mali among women who had made 3–4 antenatal visits than among those who had made at least five ANC visits.

In terms of the number of children, the results show that compared with women with seven or more children, those with one or two children (aOR = 0.59 [95%CI = 0.41–0.84] in Benin and 0.58 [CI = 0.42–0.82] in Mali) are less likely to give birth at home.

Additionally, women who often or regularly listened to the media were less likely to have homebirth compared with those who did not pay attention to the media (aOR = 0.42 [95%CI = 0.26–0.67] in Benin and aOR = 0.65 [95%CI = 0.50–0.85] in Mali for women who regularly followed the media).

## Discussion

This study performed a comparative analysis of the factors associated with homebirths in Benin and Malitwo West African countries, and the factors associated with them. Data from the 2018 DHS conducted in Benin and Mali were used. We found a difference in the proportion of home deliveries in both countries with 13.7 and 32.9% in Benin and Mali, respectively. This difference between the two countries could be explained by the availability and geographical coverage of health facilities and by the differences in health policies in place for maternal and child health. Benin in 2009 (28) and Mali in 2005 (29) developed and implemented a free cesarean section policy to increase access to emergency obstetric care for women to reduce maternal morbidity and mortality. Although this policy has improved access to care for pregnant women, it has not succeeded in eliminating homebirths in the countries, and the problems with women's access to obstetric care still exist, especially in Mali (30).

The results of our study showed that distance to the health center is an important factor influencing the place of delivery; the risk of giving birth at home was higher among women in both countries who considered the distance to the health facility to be a problem. This finding is consistent with the results of several previous studies in Africa (5, 9, 10, 12, 31–34), Peru (35), and India (36). The distance to health services certainly has a dual influence on their use: it is used as a reason for not seeking care in the first place and is a real barrier to accessing care (37). Many pregnant women do not even try to reach a facility for delivery because it is difficult to walk several kilometers during labor and impossible if labor begins at night when transport is often not available. The barrier effect of the distance is the strongest when combined with the lack of transport and poor road conditions (38).

The results of the study showed that ANC visits are associated with homebirths in a statistically significant manner. Women who did not make ANC visits were more likely to give birth at home; the more ANC visits a woman makes during her pregnancy, the less likely she is to give birth outside a health facility. This result confirms those found by Gebremichael et al. in nine sub-Saharan countries (32), Kimario et al. in Tanzania (39), Sangho et al. in Mali (40), and Paraiso et al. (41) in Benin. The significant effect of ANC underscores the role that pregnancy care plays in informing women of the benefits of institutional delivery and linking them to appropriate services (42). ANC visits has been shown to have a positive impact on the quality of life of women, and it is indeed the most favorable point of contact for mothers to obtain more information about the risks and problems they may encounter during childbirth (43).

Regarding individual characteristics, the results indicated that a woman's education level, marital status, and the number of children were statistically significant in their association with homebirth. Women with no education, those who were married, and those with seven or more children were more likely to give birth at home. This finding is also consistent with previous studies (32, 39, 42–44). The communities demonstrating high fertility may be more conservative in their attitudes toward using the service and the expected roles for women and may have lower levels of economic development, which influence a woman's ability to seek care during labor. High fertility may also reflect a lack of reproductive health services or a lack of awareness of these services if available, both of which have clear implications for the use of maternal health services (45).

Concerning the place of residence, living in a rural area was associated with a higher proportion of homebirths in both Mali and Benin. This can be attributed mainly to poorer geographical access and transport difficulties in reaching health facilities, which generally also lack adequate infrastructure for care (35).

The standard of living of households was also found to be a factor influencing the place of delivery; women from poor households are more likely to give birth at home. Affordability mainly influences whether the woman goes to a facility or not (46). Therefore, policies that waive the delivery fees and provide free delivery are likely to increase women's access to health services. In Burkina Faso, the findings of Ben-Ameur et al. (47) show that eliminating fees for facility-based deliveries benefited the poorest households and reduced inequalities of access between the poor and the rich.

The present study found that non-exposure to the media was associated with a greater likelihood of having a homebirth in both countries studied. This result is consistent with other studies conducted elsewhere in the world (48–51). The effect could be explained by the fact that most media programmes repeatedly promote institutional delivery, which may influence mothers to develop a positive behavior toward institutional delivery (43). Also, in India, Sinha et al. (48) have shown that exposure to mass media significantly increased the likelihood of receiving full ANC visits and institutional delivery in both regions. However, as found in Ethiopia, limited electricity distribution in rural areas and low levels of education may combine to reduce media exposure, denying women the benefits of media-facilitated health promotions (43).

The results of the multilevel logistic regression analysis showed that the contextual effect was significant, demonstrating that community factors influence home birth. Previous research has shown the effect of contextual and community factors on the choice of birthplace. Thus, community factors, geographical factors, availability and physical access to health services, and road conditions are the mechanisms through which community effects can manifest themselves (31). The community characteristics that had a strong direct positive influence on women's decision to seek maternity care included the percentage of women in a community who had given birth in a health facility, high standard of living in urban neighborhoods, and the presence of a health worker in the community providing antenatal care (ANC) (42, 45). The constraints on the use of maternal health services have been associated with poor road conditions, a high average number of children per woman in the community, and living far from medical care facilities (31). Furthermore, community beliefs and norms are reflected in an individual's healthcare decisions, as individual behavior is influenced by how they believe the community judges their actions (12). Community-level characteristics represent a unique social context that not only affects how individuals perceive and respond to health or other problems in the social environment but also exerts independent effects on the health outcomes of individuals in the community (34).

This study used data from the DHS, which uses standard data collection procedures to ensure reliability and a multi-stage probability sampling methodology to select clusters and households from geographic sampling frames that cover the entire territory of the participating countries. However, there are some limitations to this study. The first limitation is the cross-sectional nature of the data collected which means the outcome and explanatory variables were measured at the same time, and therefore cannot guarantee any causality of the associations. The second limitation is the recall and self-reporting bias of the information collected by the DHS.

## Conclusion

Delivery outside a health facility and unassisted by skilled health personnel may expose women to a higher risk of maternal and infant morbidity and mortality. However, more than 30 of women in Mali and over 15% of the women in Benin give birth at home, and the factors associated with homebirth are the low levels of education, residence in rural areas, lack of antenatal visits during pregnancy, distance to the health center, and individual characteristics such as marital status and the number of children. Non-exposure to the media also increases the risk of giving birth at home. Further, contextual factors related to the village residence (or area of residence otherwise) are also important in understanding why some women give birth at home.

Increasing the demand for and access to women's health services (prenatal consultations, assisted deliveries, etc.) by improving the availability and quality of services and establishing community health centers could help significantly reduce the risk of homebirths and, thus, help the fight against maternal and infant mortality. This could be achieved by bringing care closer to women through the development of community healthcare systems that duly on-board local actors, cultural values, endogenous resources, and initiatives developed and implemented by the community.

## Data Availability Statement

Publicly available datasets were analyzed in this study. This data can be found here: https://dhsprogram.com/data/available-datasets.cfm.

## Author Contributions

AB, HB, and EJ conceived the study with the contributions from EK, SP, and FD. AB analyzed the data with the contributions from HB, EJ, EK, SP, and FD. AB, EJ, HB, EK, SP, and FD for the interpretation. AB drafted the initial manuscript. All authors contributed to manuscript revision and have approved the final version.

## Conflict of Interest

The authors declare that the research was conducted in the absence of any commercial or financial relationships that could be construed as a potential conflict of interest.

## Publisher's Note

All claims expressed in this article are solely those of the authors and do not necessarily represent those of their affiliated organizations, or those of the publisher, the editors and the reviewers. Any product that may be evaluated in this article, or claim that may be made by its manufacturer, is not guaranteed or endorsed by the publisher.

## Footnotes

^1^ https://www.unfpa.org/fr/data/world-population-dashboard

^2^CPS/SSDSPF, INSTAT, INFO-STAT, Centre d'Études et d'Information Statistiques (INFO-STAT), 2014, Enquête Démographique et de Santé (EDMS-V) 2012-2013 Mali, Document Ronéotypé, USAID/ INSTAT/Coopération Canadienne, mai, p 4. http://www.worldbank.org/en/country/mali/overview, consulté le 07 décembre 2015.

## Abbreviations

aOR: Adjusted Odds ratio
ANC: Antenatal care
CI: Confidence interval
DHS: Demographic Health Survey
ECOWAS: Economic Community of West African States
EmONC: Emergency Obstetric and Neonatal Care
HDI: Human Development Index
MMR: Maternal mortality ratio
PSU: Primary survey unit
SDG: Sustainable Development Goal
WHO: World Health Organization.
